# Association of Adverse Childhood Experiences with Glycemic Control and Lipids in Children with Type 1 Diabetes

**DOI:** 10.3390/children7010008

**Published:** 2020-01-18

**Authors:** Anoop Mohamed Iqbal, Seema Kumar, Janet Hansen, Mary Heyrman, Rebecca Spee, Aida Lteif

**Affiliations:** Department of Pediatric and Adolescent Medicine, Division of Pediatric Endocrinology, Mayo Clinic, 200 First Street SW, Rochester, MN 55905, USA; iqbal.anoop@marshfieldclinic.org (A.M.I.); kumar.seema@mayo.edu (S.K.); hansen.janet@mayo.edu (J.H.); heyrman.mary@mayo.edu (M.H.); spee.rebecca@mayo.edu (R.S.)

**Keywords:** adult survivors of child adverse events, type 1 diabetes mellitus, hemoglobin A1C, dyslipidemias, obesity

## Abstract

Adverse childhood experiences (ACE) have been associated with a greater prevalence of risky behaviors and chronic health conditions, such as diabetes in adulthood. While adolescents with risk taking behaviors experience worsening of diabetic metabolic control, it is yet to be determined whether glycemic management in children and adolescents is negatively and independently influenced by ACEs. This study examines the relationship between ACEs in children and adolescents with type 1 diabetes (T1DM) and glycemic control, BMI and lipids. For such children, we hypothesized that hemoglobin A1c (HbA1c) is positively correlated with ACE scores. Parents of children (age 2–18 years) with T1DM completed a validated ACE questionnaire. The associations between parent and child ACE score and HbA1c, lipids and BMI z-scores were assessed using linear regression. The prevalence of any ACE was 27.9% among children and 49.0% among parents. HbA1c was significantly higher in children who had exposure to three or more ACEs (β: 0.63 (4.5 mmol/mol); *p* = 0.02), in those who had a parent exposed to four or more ACEs (β: 0.87 (7.2 mmol/mol); *p* = 0.03), in children who had exposure to household incarceration (β: 0.62 (4.4 mmol/mol); *p* = 0.05) and children who witnessed or had been victim of violence in the neighborhood (β: 0.71 (5.4 mmol/mol); *p* = 0.02). ACEs were highly prevalent among children with T1DM and had a positive association with glycemic control.

## 1. Introduction

Type 1 diabetes mellitus (T1DM) accounts for about two thirds of all newly diagnosed cases of diabetes among children and adolescents residing in the United States [1,2], where the incidence of T1DM is steadily rising [3]. The management of T1DM is multifaceted, and one key aspect is the adherence of the patient and family members to many disease specific behaviors. Developmental changes of the patient and alteration in family dynamics are crucial factors that influence optimal glycemic control [4].

Adverse childhood experiences (ACEs) are stressful or traumatic events that have been linked to the development of a variety of health problems, such as obesity, depression and anxiety. In a study by Randell et al., parental adverse childhood experiences also correlated positively with child adversity [5]. Parental psychological stress has been shown to be a predicator of metabolic control in children with type 1 diabetes [6]. In those children, identifying parental and personal exposure to adverse childhood events may therefore lead to implementation of early intervention aimed at optimizing family dynamics and indirectly optimizing metabolic control. It is critical that their effects be studied in children with T1DM who are already at increased risk for adverse health consequences.

There have been several studies depicting the negative effects of childhood trauma and adverse experience on health outcomes, such as suicidal ideation, depression and substance abuse [7,8,9,10,11]. There are no studies looking at the relationship between ACEs and health outcomes in children with T1DM.

The goal of this study was to explore the association between reported ACEs in participants with T1DM and their parents with respect to glycemic control, body weight and lipids. There is scarcity of data looking at those outcomes, which may lead to adult chronic health conditions known to be associated with ACEs. We hypothesized that higher ACE scores are associated with poor glycemic control, higher body mass index and higher levels of dyslipidemia.

## 2. Methods

### 2.1. Participants

All participants and their parents were evaluated in the multi-disciplinary pediatric diabetes clinic at Mayo Clinic, Rochester MN. Children and adolescents with T1DM ages 2–18 years were enrolled in this study.

### 2.2. Procedure

The information regarding the adverse childhood experiences was collected using a validated questionnaire used in the 2011–2012 National Survey of Children’s Health [12]. The questionnaire consisted of 9 questions directed at assessing abuse, neglect and household dysfunction during childhood [12]. Parents were requested to complete an electronic version of this validated adverse childhood experiences questionnaire. The parent answered one questionnaire regarding his or her own childhood experience and another one on behalf of the child. If two parents had accompanied the child, we allowed them to choose who would fill the questionnaire. HbA1c and anthropometric values were collected the same day of the survey. Fasting lipids, drawn closest to the interview date, and within 1 year of enrollment, were analyzed in order to assess the effect of ACEs on cardiovascular health outcomes. The BMI z-scores of the participants were determined using the age-specific and sex-specific median BMI, generalized coefficient of variation (S) and the power of the Box-Cox transformation (L) by the given formula: (((BMI/median BMI)^L^) − 1)/(L × S), based on the U.S. Centers for Disease Control and Prevention growth curves [13]. We also collected the medical insurance information from the medical records.

### 2.3. Measures

The occurrence of each adverse experiences in the study population was determined. The “yes” response to each question was considered as 1 point, and the sum of all responses was calculated as the total adverse childhood experiences score. We analyzed the data for association between glycemic control in children as measured by HbA1c, lipids, and weight status as measured by BMI z scores, with the individual ACE and total scores from both the parent and the child’s forms. BMI z scores, HbA1c and lipid panel results were extracted from the electronic medical records. HbA1c was measured using ion-exchange high-performance liquid chromatography (HPLC).

Abnormal lipid levels were defined according to the National Heart, Lung and Blood Institute’s (NHLBI) Expert Panel on Integrated Guidelines for Cardiovascular Health and Risk Reduction in Children and Adolescents [14] as: total cholesterol ≥ 5.172 mmol/L, HDL cholesterol < 1.03 mmol/L and non-HDL cholesterol ≥ 3.75 mmol/L. We looked at the insurance status as a surrogate marker for socioeconomic status.

This study was approved by the Institutional Review Board of Mayo Clinic, Rochester, MN, USA (number 16-002719). Records of only those patients who had given research authorization were reviewed. Written informed consent was obtained from every participant

### 2.4. Statistical Analysis

Data are expressed as means and standard deviations or counts (%) as appropriate. Adjusted odds ratio and 95% confidence intervals were obtained from multivariate logistic regression. Statistical models were used to estimate the strength of the associations of HbA1c and lipids with total adverse childhood experiences score (i.e., sum of all the “Yes” responses from the 9 questions) treated as continuous variable for the linear regression models and binary variable for the logistic regression models (child questionnaire (≥3 versus <3) and for parent questionnaire (≥4 versus <4)). We did not intentionally separate the cut off points for numbers of Adverse Childhood experiences between children and adults. The 3 or more in children and 4 or more in adults are the numbers that have been found to be associated with high HbA1c. We also ran logistic models to estimate the association of HbA1c and lipids for each of the 9 categories of adverse childhood experiences. Multivariate logistic regression models were used to compare between obese status versus non-obese status. All models were done univariately and then adjusted for age, BMI z-scores, insurance type and gender. All calculated *p*-values were two-sided and *p*-values less than 0.05 were considered statistically significant. The JMP software version 10 (SAS Institute, Inc., Cary, NC, USA) was used for all analyses.

## 3. Results

One hundred and ten children and their parents (110/121; 90.9%) had consented to participate in the study. Six participants were excluded, as their forms were incomplete. Mean age was 12.53 ± 3.86 years and 56.7% (59/104) of participants were boys. The racial distribution of the study population was as follows: whites (88%), African American (5%) and others (7%). Ninety-nine percent of the study population stated they were non-Hispanic or non-Latino for their ethnicity. Thirty-six percent (37/104) were on a continuous subcutaneous insulin infusion program. The mean HbA1c was 8.29 ± 1.35 [67 ± 8.88 mmol/mol] (Table 1). Seventy-two percent of children and adolescents had a HbA1c > 7.5% [58.5 mmol/mol], which was the 2016 American Diabetes Association (ADA) goal for children with T1DM [15]. The mean duration of diabetes for the study population was 5.2 ± 4.2 years. Hypercholesterolemia (total cholesterol ≥ 1.72 mmol/L) was seen in 10.7%, low HDL cholesterol (<1.03 mmol/L) was seen in 9.1% and high non-HDL cholesterol (≥5.172 mmol/L) was seen in 9.9%. We did not identify any participant with a diabetes-related complication, such as retinopathy and microalbuminuria; 77.9% of participants (81/104) had private insurance. There were two participants on oral contraceptives and none of the participants were on statins.

**Figure 1:**
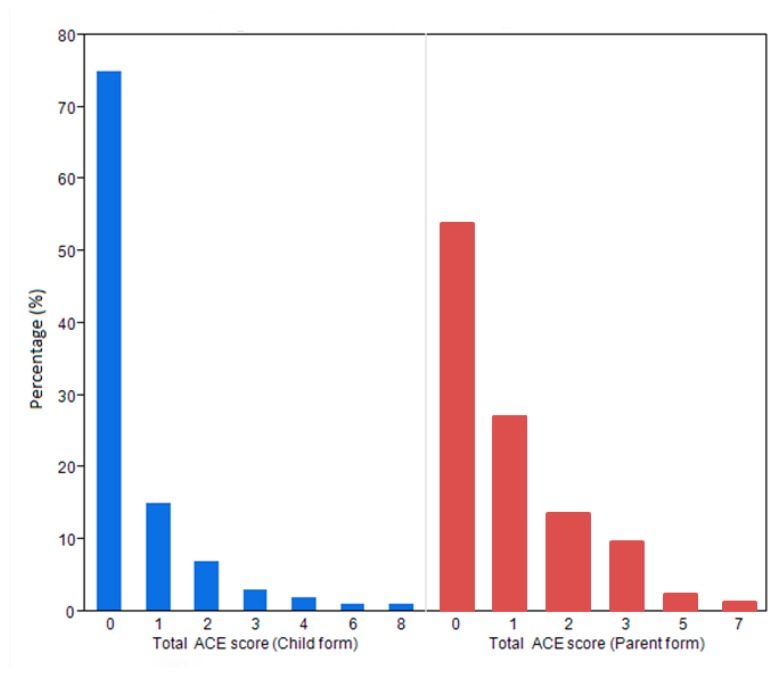
Total adverse childhood experiences (ACE) scores in children and their parents.

We found that 27.9% (29/104) of children and adolescents in the study were exposed to at least one adverse experience, while 49% (51/104) of the parents had exposure to an adverse experience during their childhood (Figure 1).

**Figure 2:**
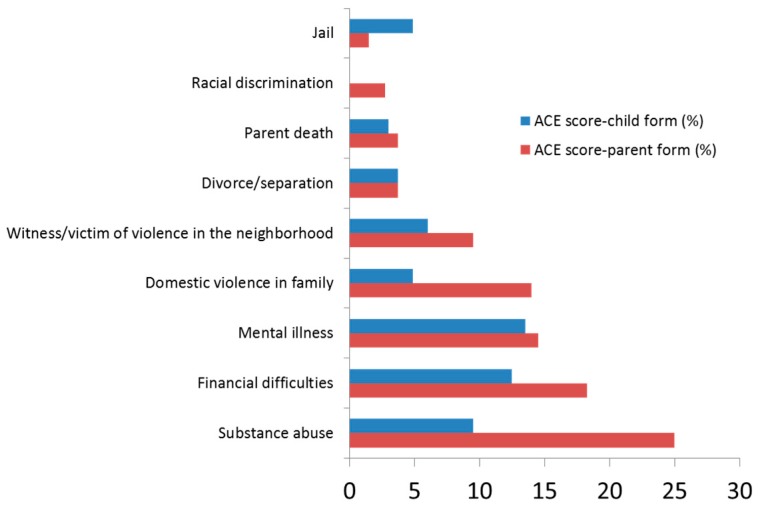
Distribution of the various childhood events.

The most common adverse experience that children were exposed to was having lived with a caregiver who was mentally ill, suicidal or severely depressed (13.46%), followed by having lived in a household where the family income was inadequate to cover the basic necessities, such as food and housing (12.5%). The most common adverse experience parents had been exposed to while they were children, was having lived with a caregiver who had problem with alcohol or drugs (25%); next was having lived in a household where the family income was inadequate to cover the basic necessities, such as food and housing (18.27%) (Figure 2).

**Figure 3:**
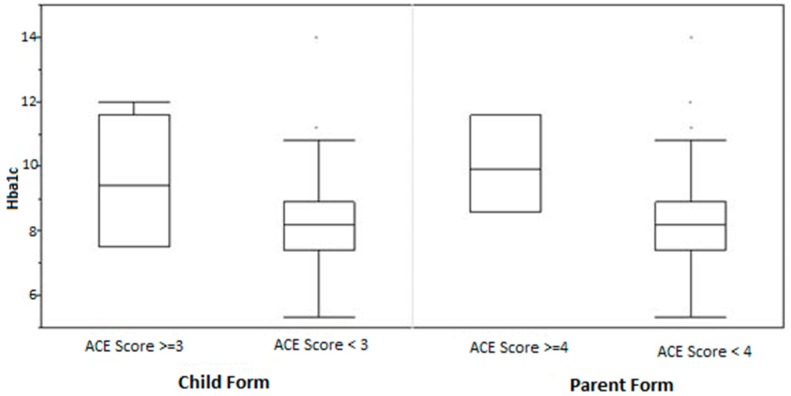
Relationship between ACE scores and HbA1c.

HbA1c was found to be significantly higher among those children who had exposure to three or more adverse experiences compared to those with exposure to less than three adverse experiences (9.66% (12.8 mmol/mol) versus 8.19% (66.0 mmol/mol); β: 0.63 (4.5 mmol/mol); 95% CI: 0.11–1.15 (1.08–10.2 mmol/mol); *p* = 0.02), after adjusting for age, gender, insurance type and BMI z-score (Table 2). We also found that HbA1c was significantly higher among children who had lived with parents who had exposure to four or more adverse experiences during their childhood compared to those with exposure to less than four adverse experiences, (10.03% (86.1 mmol/mol) versus 8.24% (66.6 mmol/mol); β: 0.87 (7.2 mmol/mol); 95% CI: 0.11–1.63 (0.96–15.5 mmol/mol); *p* = 0.03), after adjusting for age, gender, insurance type and BMI z-score (Figure 3).

When we looked at the association of HbA1c with a specific type of adverse childhood experience, and after adjusting for age, sex, insurance type and BMI z-scores, HbA1c (β: 0.62 (4.4 mmol/mol); *p* = 0.05) was significantly increased among children who lived with a household member who served time in jail. HbA1c was significantly higher among children who had witnessed or had been a victim of violence in the neighborhood (β: 0.71 (5.4 mmol/mol); *p* = 0.02), also after adjusting for age, sex, insurance status and BMI z-score.

Exposure of children to one or more ACE was associated with a lower prevalence of obesity (OR: 0.14; *p* = 0.02). Additionally, non-HDL (high density lipoprotein) cholesterol (β: 0.42 mmol/mol, *p* = 0.05) was significantly increased among children who lived with a household member having served time in jail. There was no statistically significant association between HbA1c and lipids when compared to other individual questions.

## 4. Discussion

Our study highlights the association of stressful life events among children with T1DM with poor glycemic control and dyslipidemia. Self-reported adverse childhood experiences were found in 27.9% of children with T1DM and 49% of parents. HbA1c was 0.63% [4.5 mmol/mol] higher in children who had experienced three or more adverse experiences when compared to those who had less than three adverse experiences. HbA1c was 0.87% (7.2 mmol/mol) higher in children who lived with parents who themselves had exposure to four or more adverse childhood experiences compared to those who had less than four adverse childhood experiences. The prevalence of at least one adverse childhood experience among parents was almost twice as high when compared to children (49% versus 27.9%). The most common adverse childhood experience faced by children was living with a caregiver who was mentally ill, suicidal or severely depressed (13.46%), and living with a caregiver who had problem with alcohol or drugs was the most prevalent adverse childhood experience among the parents (25%). The prevalence of exposure to at least one adverse childhood experience among children in our study was 27.9%. The 2016 National Survey of Children’s Health also showed a comparable prevalence of 25.6% and 19.8% in children with special healthcare needs (CSHCN), at the national level and in Minnesota respectively [16]. The difference in the prevalence between children and their parents is possibly related to under reporting by the parents because of fear of legal consequences, as data was being collected at a multidisciplinary clinic staffed by medical providers and a social worker. Our study was specifically looking at only children with T1DM, while the national study group comprised CSHCN with many chronic illnesses, such as diabetes, learning disability, asthma and seizure disorder.

We found that HbA1c was significantly increased among children who experienced three or more adverse events (9.66% (12.8 mmol/mol) versus 8.19% (66.0 mmol/mol); β: 0.63 (4.5 mmol/mol); 95% CI: 0.11–1.15 (1.08–10.2 mmol/mol); *p* = 0.02). Similarly, Commissariat et al. showed that among 178 teens with T1DM, glycated hemoglobin was higher in those who experienced more stressful life events [17]. We also found that the HbA1c was 0.62% (4.4 mmol/mol) higher in children who lived with a household member who had served time in jail, and 0.71% (5.4 mmol/mol) higher in those who had witnessed or had been a victim of violence in the neighborhood, when compared to those who did not have such exposure. Financial constraints which could negatively affect the management of T1DM are likely present when one parent is serving time in jail. There also may be less opportunities for parental monitoring of the patient’s adherence to treatment recommendations. In a cross-sectional study of 257 children aged 11–14 years with T1DM, Cacavale et al. showed that decreased family density (fewer adults to children in a family) was related to poorer adherence and glycemic control [4]. Witnessing or being a victim of violence in a neighborhood could also be interpreted as living in an economically deprived and socially disadvantaged neighborhood, which could lead to poorer glycemic control. The positive correlation between HbA1c in adults and adverse events they had experienced in childhood has been previously reported. A prospective longitudinal study from Ireland revealed that those adults who were raised in socioeconomically disadvantaged families and who were socially isolated had a higher risk of having dyslipidemia and elevated glycated hemoglobin [18]. Another study by Thomas et al., who surveyed 9310 45-year-old participants from the 1958 British birth cohort, also showed that household dysfunction and lack of paternal care during childhood was associated with HbA1c ≥6% [19]. Rechenberg et al. conducted a questionnaire based study involving 320 children with T1DM of ages 11–14 years, which showed those participants who underwent general stress and diabetes specific stress had significantly elevated HbA1c [20].

Our study also showed that children having lived with parents having experienced four or more adverse childhood experiences had a 0.87% (7.2 mmol/mol) higher HbA1c when compared to children of parents who had exposure to less than four adverse childhood experiences. Randell et al., in their study on 215 parents and their children, reported that those children who lived with parents with exposure to four or more adverse childhood experiences, were at higher risk of being exposed to childhood adversities [5]. Parents who had experienced significant stress may find it challenging to provide their children with secure and stable environments to thrive. Children who had exposure to at least one adverse childhood experience had lower BMI z-scores. Most of the literature suggests that exposure to adverse childhood experiences is a risk factor for developing obesity [21,22]. We speculate that the association of lower BMI z-scores with exposure to any adverse childhood experience among children with T1DM, could be caused by missing insulin doses, leading to poor glycemic control. Intentional insulin omission and eating disorder is quite prevalent among children with T1DM [23,24]. Isohookana et al. also showed that out of 449 Finnish adolescents, those who had experienced an adverse event like sexual abuse, were at risk of developing extreme weight loss behaviors. They also showed that participants who lived with unemployed parents had an increased risk of being underweight [25].

Our study showed that the non-HDL cholesterol was significantly higher among those children who had lived with a household member who served time in jail. Non-HDL cholesterol and HDL/LDL ratio have been shown to be better predictors of atherogenesis and cardiovascular risk than LDL cholesterol. Spann et al. showed a lower HDL cholesterol and HDL/LDL ratio, among 454 adults, predominantly African American, who had experienced childhood trauma [26]. Among 83 participants with schizophrenia, it was found that exposure to childhood trauma was associated with higher LDL cholesterol and blood pressure [27].

Our study has several limitations. We acknowledge that our sample size is rather small, particularly of children that had reported three or more adverse childhood experiences. We did not present demographic information regarding the parents except for type of insurance. In addition, we did not compare the demographics of the families that declined to participate to those who were part of the study. As the adverse childhood experiences were reported retrospectively by the parents, there could potentially be recall bias. There is also a possibility of under reporting of the adverse childhood experiences of children by their parents. We elected to have the parents fill out the questionnaire on behalf of their children for consistency, since the younger participants may not have been able to read and comprehend the questions. We did not separate the effect of adverse childhood experiences based on the parent’s gender. We also did not have data on demographics of parents, parent marital status, dietary history and physical activity records. As the study was conducted within one geographical area and data was collected at one point, it limits the generalizability of the findings. Finally, even though our study demonstrates associations between adverse childhood experiences and glycemic control, weight status and dyslipidemia, causality cannot be ascertained.

## 5. Conclusions

The results of our study suggest that adverse childhood experiences are prevalent among children with T1DM. We found that adverse childhood events experienced by a child and their parents had negative impact on the glycemic control. Adverse childhood experience assessment may play a future role in the routine psycho-social evaluation of children with T1DM. Those individuals with exposure to adverse childhood events could possibly benefit from more intense psychological interventions which may improve their overall health outcomes.

## Figures and Tables

**Table 1:** Demographics and laboratory characteristics of study subjects.

	Mean	Standard Deviation
Age (years)	12.53	3.86
Gender Male 59 (56.7%)		
Weight (kg)	53.79	21.48
Height (cm)	152.78	20.40
BMI (kg/m^2^)	22.01	5.13
BMI z-score	0.84	0.93
HbA1c (%)	8.29	1.35
Cholesterol (mg/dL)	164.96	30.75
HDL cholesterol (mg/dL)	58.31	12.62
Non-HDL cholesterol (mg/dL)	106.22	31.07

**Table 2:** Comparison of ACE score and laboratory parameters.

	ACE Score ≥ 3 (Child Form)			ACE Score ≥ 4 (Parent Form)		
	Estimate	CI95%	*p*-Value ^#^	Estimate	CI95%	*p*-Value ^#^
HbA1c (%)	0.63	0.11–1.15	0.02 *	0.87	0.11–1.63	0.03 *
Total Cholesterol (mg/dL)	10.10	−3.17–23.37	0.13	−7.66	−25.98–10.67	0.41
HDL Cholesterol (mg/dL)	0.26	−4.83–5.35	0.92	1.25	−5.69–8.19	0.72
Non-HDL cholesterol (mg/dL)	10.23	−3.33–23.79	0.14	−8.74	−27.39–9.92	0.35

* *p*-value < 0.05; ^#^ adjusted for age, gender, socio-economic status and BMI z-scores.
